# Demographic and Socioeconomic Factors Influencing Public Attitudes Toward a Presumed Consent System for Organ Donation Without and With a Priority Allocation Scheme

**DOI:** 10.1097/MD.0000000000001713

**Published:** 2015-10-23

**Authors:** Makmor Tumin, Khaled Tafran, Muzalwana Abdul Talib @ Abdul Mutalib, NurulHuda Mohd Satar, Saad Mohd Said, Wan Ahmad Hafiz Wan Md Adnan, Yong Sook Lu

**Affiliations:** From the Department of Administrative Studies and Politics (MT); Institute of Research Management and Monitoring (KT); Department of Applied Statistics (MATAT); Department of Economics (NMS, SMS); Department of Medicine (WAHWMA); and University of Malaya, Lembah Pantai, Kuala Lumpur, Malaysia (YSL).

## Abstract

The influence of demographic and socioeconomic factors on the public's attitude towards a presumed consent system (PCS) of organ donation was estimated in 2 scenarios: without and with a priority allocation scheme (PAS). Self-administered questionnaires were completed by 775 respondents. Using multiple logistic regressions, respondents’ objections to donating organs in both scenarios were estimated. In total, 63.9% of respondents would object to donating under a PCS, whereas 54.6% would object under a PCS with a PAS. Respondents with pretertiary education were more likely to object than were respondents with tertiary education, in both the first (adjusted odds ratio [AOR] = 1.615) and second (AOR = 1.728) scenarios. Young respondents were less likely to object than were middle-aged respondents, in both the first (AOR = 0.648) and second (AOR = 0.572) scenarios. Respondents with mid-ranged personal monthly income were more likely to object than were respondents with low income, in both the first (AOR = 1.994) and second (AOR = 1.519) scenarios. It does not seem that Malaysia is ready to implement a PCS. The educational level, age, and income of the broader public should be considered if a PCS, without or with a PAS, is planned for implementation in Malaysia.

## INTRODUCTION

Malaysia suffers from a chronic shortfall in organs for transplantation. Its organ-donation rates are low, at 0.5 and 1.87 donations per million people (PMP) in 2013 for deceased and living donations, respectively.^1^ As of December 2014, >18,000 patients in Malaysia were awaiting kidney transplants,^2^ whereas on average, only 26 transplants (sourced from deceased donors) have been performed yearly over the past decade.^3^ As living donation has led to organ trading and tourism around the world, enhancing deceased donation rates seems to be the only efficient remedy to address this shortfall in organs. The Declaration of Istanbul states firmly that the “therapeutic potential of deceased organ donation should be maximized [and] efforts to initiate or enhance deceased donor transplantation are essential to minimize the burden on living donors.”^4^

There is evidence that a presumed consent system (PCS), in which everyone is a donor unless he or she objects during his or her lifetime, yields higher rates of organ donation than an informed consent system (ICS), in which only those who registered during their lifetime are considered for organ donation.^5–8^ Some policy analysts have argued that the increase in deceased donation rates achieved in some countries after implementing a PCS actually results from the successful organization of the donation process.^9,10^ In fact, some countries have not reported any substantial increase in deceased donation rates after shifting from an ICS to a PCS. In Chile, for instance, average deceased donations declined from 8.08 PMP (2004–2008) to 6.78 PMP (2009–2013) after implementing a PCS in 2009.^1^ Nevertheless, PCS are increasingly gaining ground. Starting in 2015, a PCS will take force in Wales, with a similar transition expected in Northern Ireland.^9^

Some people might be willing to receive an organ transplant, should they need it, and yet be unwilling to donate their own organs upon their deaths.^11^ This behavior biases the equal allocation of organ transplantation among willing and unwilling people, a bias that can be minimized by giving those who want to donate a preferred position on the waiting list for an organ transplant, should they need it, over those who do not want to donate. Recently, Israel—which adopts an ICS—introduced a priority allocation scheme (PAS), granting registered donors and their families preferred positions on the waiting lists for organ transplantation. This strategy has significantly increased the number of registered donors in Israel.^12,13^

Demographic and socioeconomic factors have been associated with attitudes toward organ donation. In Malaysia, a survey showed that about 34% of Malaysians are willing to donate their organs upon death.^14^ The same study reported a significant association between ethnicity and willingness to donate.^12^ Furthermore, a study in Europe^5^ and 2 in Malaysia^11,14^ have found that willingness to donate is positively associated with higher levels of education. Moreover, personal income was found to have an inverse association with willingness to donate organs after death in Malaysia.^14^ Nevertheless, previous studies in Malaysia have not taken into account the legislative system—that is, PCS versus ICS—when analyzing people's willingness to donate organs. Thus, no study has yet estimated the correlations between Malaysians’ attitudes toward organ donation and demographic and socioeconomic factors if a PCS was to be implemented.

The main objectives of this study are to investigate:

1. The public attitudes toward a PCS and toward a PCS with a PAS, along with any differences between these attitudes.
2. The influence of demographic and socioeconomic factors on public attitudes toward deceased organ donation under a PCS and a PCS with a PAS for organ allocation.

## METHODS

To meet these research objectives, a set of questionnaires was prepared in a collaboration between social scientists and medical experts. Initially, 75 questionnaires were distributed as a pilot study. Based on the analyzed results, we made some modifications, producing a revised questionnaire. The questionnaire was self-administered, and we anticipated a lack of knowledge among the public regarding the legislative systems of organ donation. Therefore, we provided respondents with a written explanation of both systems, PCS and ICS.

To assure high-quality responses and a high response rate, our enumerators were trained to explain to respondents the importance of the study for the future of national health. All respondents were assured that their responses would be treated with complete confidentiality and used only for research purposes.

Based on previous research in Malaysia regarding organ donation,^11,15^ we expected that the probability of objection to donating organs would be about 0.5. Malaysia's population comprises roughly 15 million adults aged 18 to 65 years.^16^ Therefore, for a confidence level of 95% and a 5% precision level, a sample of 400 respondents was needed to meet the study objectives.^17^ Although a bigger sample size is always preferable, the investment required in both money and time for data collection is a concern for all researchers.^17^ Therefore, given available resources, we distributed 900 questionnaires of which 775 were completed and returned, a response rate of 86.1% (Table 1). The study was conducted from October to December 2013. The public were approached in 4 types of locations: 5 restaurants; 2 shopping malls; 1 university campus; and 3 housing areas. The social and demographic characteristics of the respondents (Table 1) resemble the overall Malaysian population as measured by the latest official census.^16^

**TABLE 1:**
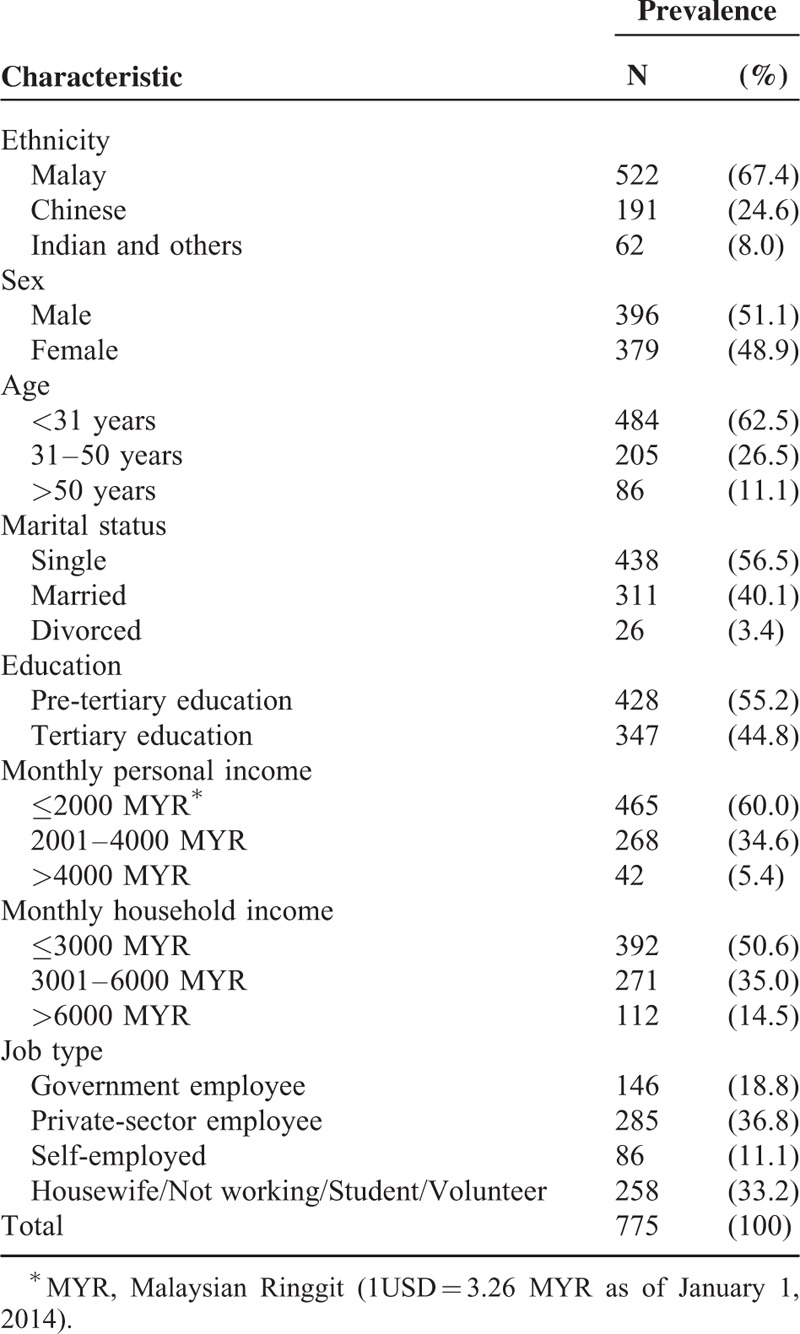
Respondents’ Profile

The questionnaire first asked respondents for their demographic and socioeconomic information. Then, they were presented with the following question: “If the Malaysian government implements the policy of PCS in which you are automatically registered as a deceased donor, would you sign the form to register your objection?” The questionnaire gave respondents 2 mutually exclusive options: “Yes, I will sign the form to register my objection”; and “No, I will not sign the objection form.” Next, respondents were presented with following question: “Would you still want to sign the form declaring that you refuse to donate your organs if the government were to implement a policy stating that you would then not be given priority to receive an organ in the future (should you need it)?” Respondents were given 2 options from which to choose: “Yes, I would still want to sign the form to register my objection”; or “No, I would not sign the form.”

Several statistical analyses were performed using SPSS 20.0 (SPSS Inc, Chicago, IL). During the first stage, a bivariate analysis (Pearson *χ*^2^ test) tested the association between “objection to donating organs” (dependent variable) and several demographic and socioeconomic factors (independent variables). In the next stage, the significantly associated demographic and socioeconomic variables were regressed against the dependent variable by applying a multiple logistic regression. A 5% significance level was used as the rejection criterion for the independent variables. We performed this statistical technique for 2 models. The first model was used as a dependent variable respondents’ objection to donating organs under a PCS without a PAS, whereas the second model was used as a dependent variable respondents’ objection to donating organs under a PCS with a PAS. If a respondent would object to donating organs, the dependent variable was assigned a value of “1,” whereas the value “0” was assigned wherein a respondent stated that he or she would not object to donating organs after death. To avoid overfitting estimates, we regrouped some of the subgroups of the independent variables to assure a minimum of 10 outcome events per predictor variable,^18,19^ as follows. The “Other” ethnic group (n = 5) was merged with Indians (n = 57). Respondents with no formal education (n = 8) or primary education (n = 17) were merged with secondary education (n = 403) in one group tagged “pre-tertiary education.” Finally, volunteers (n = 7) were added to the housewife/not working/students group (n = 251).

All human studies were reviewed by the University of Malaya Research Ethics Committee (Reference Number: UM.TNC2/RC/H&E/UMREC-35). All respondents gave their informed consent prior to their inclusion in the study.

## RESULTS

Table 2 reports the breakdown of respondents’ objections to donating organs under a PCS without and with a PAS. Overall, 36.1% of respondents recorded no objection under a PCS. The remaining 63.9% originally stated that they would refuse to donate organs if a PCS was to be implemented in Malaysia. The overall rate of objection then declined to 54.6% after respondents considered the scenario in which a PAS for organ transplant would be implemented along with a PCS.

**TABLE 2:**
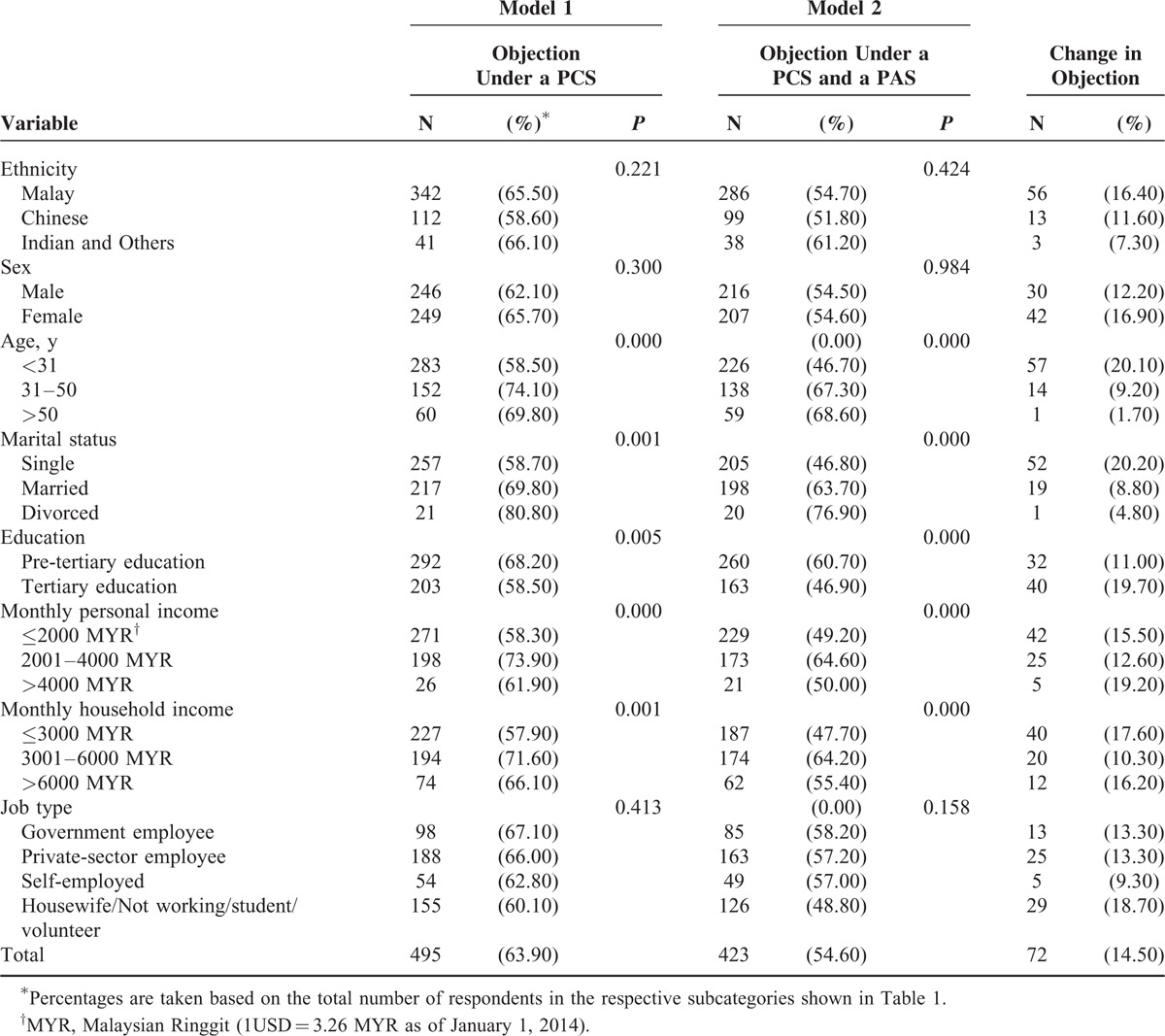
Respondents’ Objections to Deceased Organ Donation Under a PCS, With and Without a PAS, Characterized by Demographic and Socioeconomic Factors; Bivariate Analyses

The last column of Table 2 reports the reduction in objections to organ donation by demographic and socioeconomic characteristics. Declines in objections may be observed in all subcategories. Bivariate analysis reveals that age, marital status, education, monthly personal income, and monthly household income are significantly associated with objection to donating organs in both models (*P* < 0.05).

Table 3 reports the results of the logistic regressions for respondents’ attitudes toward organ donation in both models. In the first model (without a PAS), respondents with pretertiary education were approximately one and a half times more likely to object to deceased donation than were respondents with tertiary education (adjusted odds ratio [AOR] = 1.615, *P* < 0.01). In the second model (with a PAS), the differences in objections between respondents with pre-tertiary and those with tertiary education were also statistically significant, but with a slightly higher odds ratio (AOR = 1.728, *P* < 0.01).

**TABLE 3:**
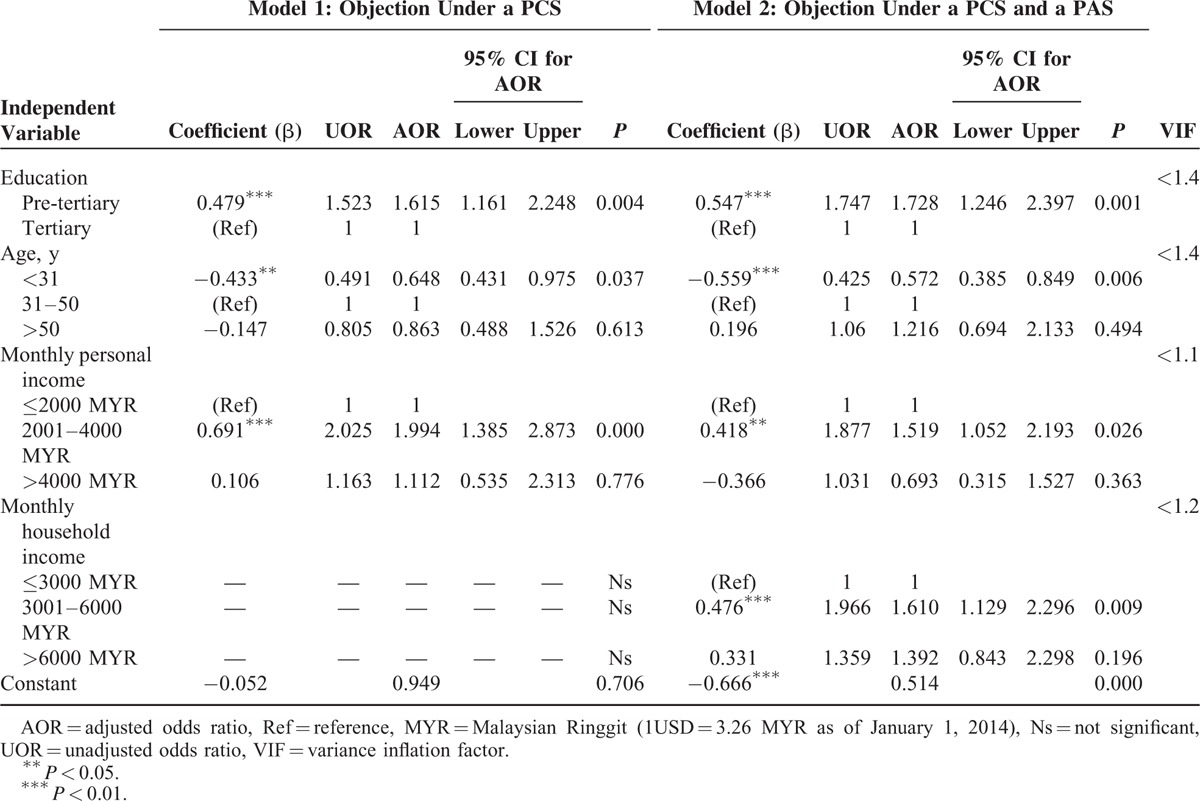
Demographic and Socioeconomic Predictors of Objection to Deceased Organ Donation; Multiple logistic Regressions

In the first model, young respondents (31 years or younger) were about 35% less reluctant to donate organs upon their deaths than were middle-aged respondents (31–50 years) (AOR = 0.648, *P* < 0.05). The same result applied to the second model, but with a lower odds ratio (AOR = 0.572) and more robust significance level (*P* < 0.01).

Without a PAS, respondents with mid-range monthly personal income (2001–4000 MYR) were twice as likely to object than were respondents with low personal income (≤2000 MYR); (AOR = 1.994, *P* < 0.001). With a PAS, middle-income respondents were about 1.5 times more likely to object to organ donation than were lower-income respondents (AOR = 1.519, *P* < 0.05).

Monthly household income was only a significant predictor of objection in the second model, in which respondents with mid-range monthly household incomes (3001–6000 MYR) were 1.6 times more likely to object to deceased donation than were their counterparts with lower household incomes (AOR = 1.610, *P* < 0.01; see Table 3). Marital status and job type were not significant predictors of objection to donating organs in either model. Finally, the low value of the variance inflation factor indicates that the models are free from collinearity (Table 3).

## DISCUSSION

### Implementing a PCS

The majority of recent studies suggest that a PCS yields higher donation rates than an ICS.^5–8^ However, most if not all of these studies concerned the developed world. By contrast, experience with PCSs in the developing world is not encouraging. For instance, Brazil abolished its PCS after noting lower donation rates and high rates of public objection under the system.^20^ Chile adopted a PCS, in 2009, and the average deceased donations PMP declined over the subsequent years.^1,21^ These cases suggest that the success of the PCS model in many developed countries, such as Spain, may be attributed not only to the PCS, but also to the well-organized donation process.^9,10^ However, the failure in the developing world to achieve higher donation rates under a PCS has been widely attributed to the absence of public trust in the medical system and the absence of a well-established donation and transplant infrastructure.^20,21^

Malaysia has used an ICS since 1974,^22^ and its donation rates are among the lowest in the world.^1,15,23^ Calls to implement a PCS in Malaysia to increase deceased donations have recently gathered attention and momentum.^24^ However, our results show that about two-thirds of the Malaysian public would object to donating under a PCS, a figure that does not encourage a transition toward this system. The picture becomes even gloomier if family consent were to be required before procuring organs from potential donors under a PCS. Another study in Malaysia found that about 70% of the families of eligible Malaysian deceased donors refused to consent to their relatives’ deceased donation.^25^ In addition, the Chilean experience indicates that family refusal may be expected to increase after shifting from an ICS to a PCS.^21^

As mentioned above, global experiences have given rise to 2 more considerations that are vital to address for the successful implementation of a PCS. First, public trust in medical systems and government institutions is essential. One study found that such a lack of trust accounts for 30% of the Malaysian public's refusal to register as deceased donors.^15^ Second, a well-established infrastructure to host donation and transplant activities is required. Lela Yasmin Mansor, the head of the Malaysia National Transplant Resource Center, stated recently that Malaysian infrastructure is not ready for a transition toward a PCS.^24^ These are 2 barriers making it more difficult for a PCS to succeed in Malaysia, so such a transition may not be the right step at the present time.

However, implementing a PAS for organ transplants may increase deceased donations.^26^ Our results suggest that such a scheme in Malaysia could reduce the general negative attitude toward organ donation (see Table 2). As in Israel, an allocation scheme could be applied under the ICS currently used in Malaysia.^12,13^ Notwithstanding the evidence from Israel that this could increase rates of deceased donations, the expected implications of such a step in Malaysia require further investigation.

### The Influence of Social Characteristics

Guy and Aldridge^27^ argued that a successful marketing campaign for organ donation requires a good understanding of the social factors that may influence donation decisions. Hence, it is imperative to understand the public's potential reactions toward a proposed organ-donation policy given various demographic and socioeconomic characteristics. Recent studies in Malaysia have found that willingness to donate organs is positively associated with educational level and age, but negatively with personal income^11,14^; however, these studies did not consider the legislative setting. Studies in the United States and Europe found that positive attitudes toward deceased organ donation are associated with higher educational level, age, and income.^5,28,29^ Our results here show that objection to donating organs under a PCS with and without a PAS is negatively associated with higher educational level and age and positively associated with personal income for those earning <4000 MYR (≈1226 USD).

Under a PCS, our findings indicate that ethnicity, sex, and job type are not significant predictors of people's attitudes toward deceased organ donation. Similarly, studies in China, Turkey, and Germany found that sex does not correlate with attitudes toward deceased donation.^30–32^ Without accounting for legislative setting, however, some studies have found that ethnicity, sex, and job type are associated with people's attitudes toward deceased organ donation in Malaysia^11,14,33^ and in other countries.^28,29,34,35^ These different findings imply that the demographic and socioeconomic predictors of people attitude toward organ donation may differ when considering the legislative settings of organ donation.

Earlier studies suggested that the Malaysian public's attitudes toward organ donation should be improved through educational campaigns that enhance awareness of organ donation and increase the public's trust in the medical system and in government institutions.^15,23^ The results of this study suggest that if a PCS was to be implemented in Malaysia, such educational campaigns should foremost target people with lower educational levels, age 31 to 50 years, and middle incomes.

### Limitations

The sample in this study was only drawn from Kuala Lumpur and its suburbs. However, we believe that this area, as a focal metropolitan region that attracts people from all Malaysian states, mirrors, by and large, the demographic profile of the Malaysian population. Similar to other studies in this area of research, association between variables does not necessarily establish causation. Future studies using a time-variant sample may be able to establish causality.

## CONCLUSION

Implementation of a PCS in Malaysia would face substantial public objection to donation, primarily from people with pretertiary education, age 31 to 50 years, earning a middle income. The large rates of objection, combined with low rates of family consent, low public trust in the medical system, and absence of an adequate donation, and transplant infrastructure, suggest that negative consequences would result from implementing a PCS in Malaysia at present. An alternative option in the short run would be to reduce widespread negative attitudes to donation through educational campaigns targeting the public at large and particularly the highest-objection groups (people with pretertiary educations, age 31 to 50 years, and middle income). An announcement of a PAS for organ transplantation would also help alleviate the public's negative attitudes toward organ donation. Such interventions may pave the way for the implementation of a PCS in the medium or long run.
